# The evaluation of a better intubation strategy when only the epiglottis is visible: a randomized, cross-over mannequin study

**DOI:** 10.1186/s12871-018-0663-9

**Published:** 2019-01-10

**Authors:** Tzu-Yao Hung, Li-Wei Lin, Yu-Hang Yeh, Yung-Cheng Su, Chieh-Hung Lin, Ten-Fang Yang

**Affiliations:** 1 Department of Biological Science and Technology, College of Biological science and Technology, National Chiao Tung University, NO.75 Po-Ai Street, Hsinchu, 30068 Taiwan; 2 Department of Emergency Medicine, Zhong-Xing branch, Taipei City Hospital, Taipei, Taiwan; 3 CrazyatLAB (Critical Airway Training Laboratory), Taipei, Taiwan; 4 Department of Emergency, Shin Kong Wu Ho-Su Memorial Hospital, Taipei, Taiwan; 5 School of Medicine, Fu Jen Catholic University, New Taipei City, Taiwan; 6 School of Medicine, Tzu Chi University, Hualien, Taiwan; 7 Department of Emergency, Dalin Tzu Chi Hospital, Buddhist Tzu Chi Medical Foundation, Chiayi, Taiwan; 8 Graduate Institute of Medical Informatics and Cardiology, Taipei Medical University, Taipei, Taiwan

**Keywords:** Difficult airway, Intubation technique, Stylet shapes, Lifting of epiglottis, Bend angles, Cormack-Lehane grade

## Abstract

**Background:**

The Cormack-Lehane (C-L) grade III airway is considered to be a challenging airway to intubate and is associated with a poor intubation success rate. The purpose of this study was to investigate whether the holding position, shapes, bend angles of the endotracheal tube (ET) and the stylet-assisted lifting of the epiglottis could improve the success rate of intubation.

**Methods:**

Thirty-two participants, 26 physicians, 2 residents, and 4 nurse practitioners, with 12.09 ± 5.38 years of work experience in the emergency department and more than 150 annual intubation events, were enrolled in this randomized, cross-over mannequin study. We investigated the effects of straight-to-cuff ET shapes with 35° and 50° bend angles, banana-shaped ET with longitudinal distances of 28 cm and 26 cm, two methods of holding the ET (either on the top or in the middle), and lifting or not the epiglottis, on the intubation duration, its success rate, and its subjective difficulty. The aim of the study is to provide optimized intubation strategies for difficult airway with C-L IIb or III grades, when the inlet of the trachea cannot be visualized.

**Results:**

The two groups that lifted the epiglottis using the stylets, in bend angles of 35° and 50°, had the shortest duration of intubation (23.75 ± 14.24 s and 20.72 ± 6.90 s, hazard ratios 1.54 and 1.85 with 95% confidence intervals [95% CI] of 1.01–2.34 and 1.23–2.78, respectively) and a 100% success rate in intubations. In the survival analysis, lifting of the epiglottis was the only significant factor (*p* < 0.0001, 95% CI 1.34–2.11) associated with the success rate of intubation.

**Conclusions:**

The use of the epiglottic lift as an adjunctive technique can facilitate the intubation and improve its success rate without increasing procedure difficulty, in C-L III airway, when only the epiglottis is seen.

**Trial registration:**

ClinicalTrials Registry (https://clincaltrials.gov, identifier NCT03366311).

**Electronic supplementary material:**

The online version of this article (10.1186/s12871-018-0663-9) contains supplementary material, which is available to authorized users.

## Background

**Fig. 1:**
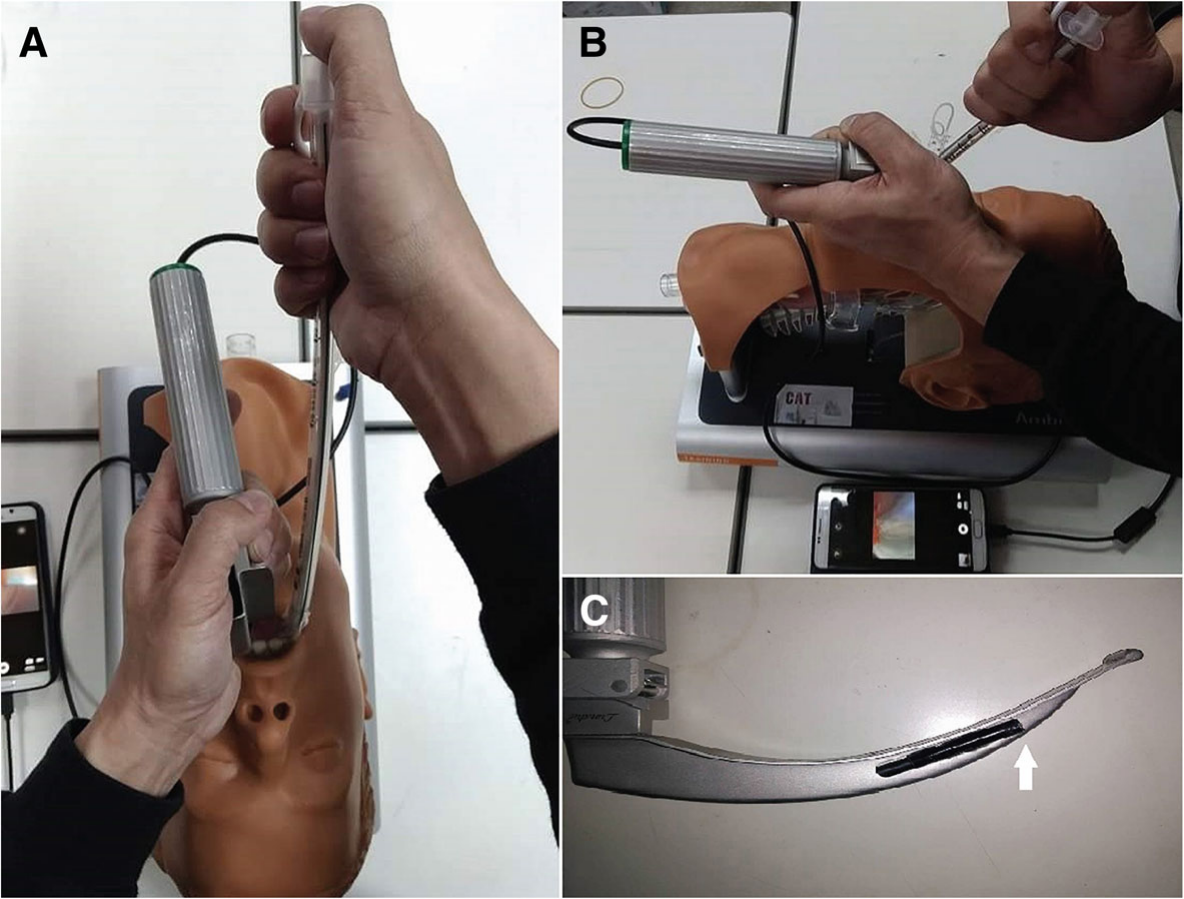
**a** Holding the top of the endotracheal tube and (**b**) bending the elbow to the chest right after the cuff passed through the incisors to elevate the tip of the tube with greater torque. All processes were recorded with a video camera. (**c**) The camera was attached to the blade for recording purposes. The participants used the direct laryngoscope without watching the screen. The white arrow indicates the location of the camera

When the vocal cords are visible using the laryngoscope, such as in C-L grades I and IIa, successful intubation is usually anticipated. However, among C-L grades IIb–IV, blind intubations (without seeing the endotracheal tube pass through the vocal cords) are more difficult because the distances between the tip of the ET and the epiglottis or other landmarks are obscured [1, 2]. Under such circumstances, if the practitioner can keep the tip of the ET moving along and just beneath the epiglottis (anterior part of the larynx), the ET would slip from the larynx into the trachea. Compared with holding the middle part of the ET and hooking an imaginary target - the tracheal inlet – that is outside of the visual field, two techniques might help. In the first method, managing and using the stylet as a Trachway intubating stylet (Biotronic Instrument Enterprise Ltd., Tai-Chung, Taiwan) could be of benefit. This involves holding the top of the ET with the right hand so that the ET can be levered immediately after the cuff passes the incisors, with the elbow bending to the chest. Such a technique may provide greater torque with which to hook and move the tip of the ET inferiorly and parallel to the epiglottis (Fig. 1a & b). This technique was initially introduced by James Ducanto (https://videopress.com/v/9dd9jIfN). The second method involves lifting the epiglottis with the tip of the stylet-equipped ET. We always use our left hands to manipulate the laryngoscope to obtain a better glottic view. When the glottic view is insufficient to perform intubation, we adjust the tip of the laryngoscope in the vallecula and try to lift more. However, Wasa et al. reported on a patient’s case that lifting the epiglottis with the stylet may also improve the glottic view directly [3].

Levitan et al. proved that the shapes and bend angles of the stylet affect the success of intubation [4]. Although a hockey shape is widely used in emergency practice, the ways in which the different stylet shapes and curvatures might affect intubation remained unclear. This study was designed to optimise intubation strategies for difficult airway with higher modified C-L grades, namely, IIb or III, when the inlet of the trachea cannot be visualized.

## Methods

### Study design and participants

This study was approved on October 31st, 2017 by the Institutional Review Board (approval no.: TCHIRB-10610108-W) and registered in the ClinicalTrials Registry (https://clincaltrials.gov, identifier NCT03366311). This study was a randomized, cross-over study. The 32 participants were attending physicians, residents, and nurse practitioners (26, 2, and 4, respectively), having 12.09 ± 5.38 years of practice in the emergency department with more than 150 annual intubation events and at least three years of clinical practice in the emergency department with more than 150 intubations. They were recruited from multiple medical centres in north and south Taiwan (Taipei City Hospital, Shin Kong Wu Ho-Su Memorial Hospital, Dalin Tzu Chi Buddhist Hospital, Shuang Ho Hospital, Taipei Municipal Wanfang Hospital, Chang Gung Memorial Hospital) during an airway training course (CATLAB, Critical Airway Training Laboratory). All participants were neither experienced in holding the top of the ET nor lifting the epiglottis with a stylet while intubating in their past practice. To successfully participate the study, they were instructed to perform these two methods and to complete at least three successful intubations on the AMBU Mannequin Intubation Airway (Ambu Enterprise Ltd., Ballerup, Denmark) before the study began.

### Protocol

**Fig. 2:**
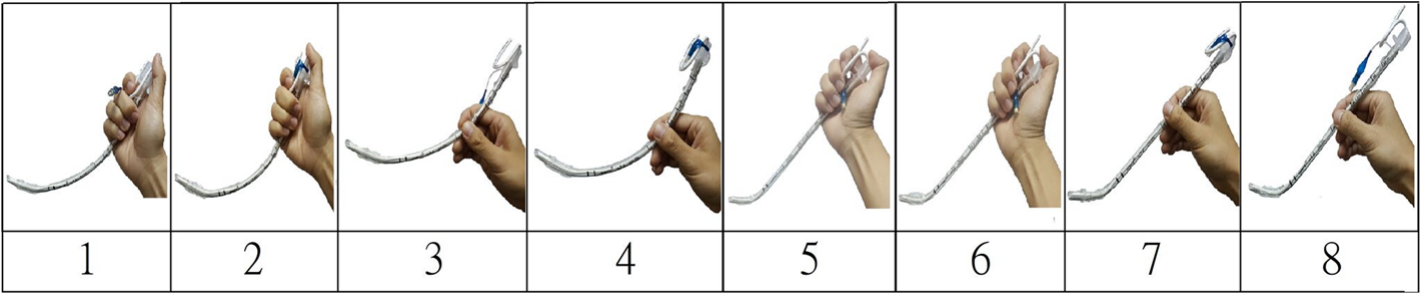
Eight settings in the study including different shapes of stylet, stylet bend angles, holding the endotracheal tube on the top or in the middle were demonstrated

A direct laryngoscope (Macintosh #3 blade) was used in all the participants, and a conventional 7.0-mm internal diameter tracheal tube (Covidien, Mallinckrodt Pharmaceuticals Ltd., Surrey, United Kingdom) was used to intubate the mannequin without assistance. They were asked to lift the epiglottis with moderate force to avoid visualization of the vocal cord and intubate under C-L grade III (only the epiglottis is visible). All intubation processes were recorded and reviewed later to ensure the intubation processes were C-L grade IIb to III with a camera embedded on the blade (Fig. 1c). The full length of the Covidien Cuffed Mallinckrodt™ ET (7.0-mm internal diameter) from the tip to the adaptor was 32.5 cm. The estimated curvature with a longitudinal distance of 28 and 26 cm was 1/0.07 (*ρ* = 0.07 m) and 1/0.08 (*ρ* = 0.08 m), respectively. These curvatures were approximate to the bend angles of straight-to-cuff of 35° and 50° at the cuff level. When the practitioners had a proper view of the glottis under direct laryngoscopy, they would receive the ET equipped with standardized stylet shapes and curvatures (straight-to-cuff 35° or 50°; banana shape with a longitudinal distance of 26 cm and 28 cm) and be instructed to hold the ET on top or in the middle (Two different shapes with each shape of two different curvatures and two different holding positions formed 8 possible settings (Fig. 2). These were randomized using random.org before the study began, (https://www.random.org/lists/). The participants only knew the shape at the time when the glottic view fulfilled the study setting and were asked to keep the shape and holding position during the intubation. Intubation time exceeding 90 s was considered as failed attempt. A successful intubation was defined as one when the tube was passed through the vocal cords exceeding the vocal cords marker. After each intubation, the participants provided a subjective visual analogue scale (VAS) score to assess the difficulty (0, not difficult; 10: impossible to intubate). All the study processes were recorded by a video camera on the tip of blade (Fig. 1c). The time from inserting the laryngoscope in the mouth of the mannequin, the time of initial proper glottic view to initiate tracheal intubation, the time of withdrawal of the endotracheal tube, and success or failure of intubation were all determined retrospectively by viewing the video clips.

At the end of the study, the participants performed intubations by lifting the epiglottis and holding the middle part of the stylet-equipped ET, straight-to-cuff shape of 35° or 50°, in a randomized order (two possible settings with random.org, https://www.random.org/lists/) (Additional file 1: Video S1). All practitioners intubated the mannequin a total of ten times.


Additional file 1: **Video S1.** The video clip shows the technique of epiglottis lifting under direct laryngoscope. (MPG 570 kb)


### Measurements

The number of years of experience in the ED, the specialty of the participants (attending physicians, residents, or nurse practitioners), and height of the participants were all recorded. The primary outcome in our study was the total duration of the intubation process (laryngoscope blade in and out of the mouth of the mannequin) and intubation success rate. The secondary outcome was subjective difficulty scales. All the results were reviewed and calculated based on the recorded video clips and VAS records.

### Statistical analysis

Study data was taken by convenience sampling from our airway training courses (CATLAB). The characteristics of the participants, glottis views during intubation, duration of intubation, success rate of intubation, and VAS scores were collected and analyzed in the study. We evaluated the time to successful intubation by plotting Kaplan-Meier survival curves to determine the trends. To cope with the correlated data from multiple intubation attempts by the same participants, we applied Cox regression models stratified by proportional hazard types. We used these models in the evaluation of the different manipulations of the hazard ratios (HRs) of successful intubation after adjusting for relevant confounding factors, namely duration of service in years, sex, height, type of participant, and different shapes and bend angles of the stylet.

Linear regression was used to evaluate intubation time and VAS score, while logistic regression was applied to evaluate the odds ratio of the success rate by using the VAS scores after adjusting for the confounding factors. The generalized estimate equation method was adapted to account for the clustering of the participants.

SAS statistical package version 9.4 (SAS Institute, Inc., Cary, NC, USA) and STATA version 11.2 (StataCorp, College Station, TX, USA) were used for data analysis. A two-tailed *p* value of < 0.05 was considered statistically significant.

## Results

Table 1 shows the distribution of sex, class, height, and duration of practice in the emergency department of the 32 participants. Of the 32 participants, 26, 4, and 4, were attending physicians, residents, and nurse practitioners, respectively. After the video clips were reviewed, 96.87% of glottis views were C-L grade III, and the rest were grade IIb. The overall success rate of the intubation was 95.63%. The duration of the intubation with holding top of banana shape stylets with 28 cm and 26 cm longitudinal distance, holding middle of banana shape stylets with 28 cm and 26 cm longitudinal distance, holding top of straight-to-cuff with 35 degrees and 50 degrees bend angles, holding top of straight-to-cuff with 35 degrees and 50 degrees bend angles were 33.63 ± 33.87 s, 31.91 ± 31.82 s, 30.10 ± 18.73 s, 31.06 ± 24.59 s, 34.81 ± 27.70 s, 28.03 ± 16.36 s, 34.10 ± 32.12 s, 27.53 ± 16.31 s, 23.75 ± 14.24 s, 20.72 ± 6.90 s, respectively; the success rate were 90.63, 93.75, 96.88, 93.75, 93.75, 96.88, 93.75, 96.88, 100, 100%, respectively; the VAS scores were 4.24 ± 2.11 cm, 4.34 ± 2.33 cm, 4.62 ± 1.94 cm, 4.48 ± 2.12 cm, 4.67 ± 2.30 cm, 4.36 ± 2.32 cm, 4.29 ± 4.48 cm, 3.90 ± 2.12 cm, 4.15 ± 2.22 cm, 3.97 ± 2.13 cm, respectively (Table 2).

**Table 1:** Participant characteristics

Variable		Mean		Standard deviation
Age (years)		38.56	±	5.19
Duration of Practice in the emergency department (years)		12.09	±	5.38
Total	32			
Attending physician	26			
Resident	2			
Nurse practitioner	4			
Height (cm)		169.69	±	6.47
Men:women	27:5

**Table 2:** Ten different subgroup settings and the results of the duration of intubation, success rate, and visual analogue difficulty scoring

	Manipulation Holding part of tube	Shape	Longitudinal distance (cm) Bend angles (°)	Duration of intubation			Success rate (%)	Visual analogue scale score (cm)
				Mean ± Standard deviation (seconds)	HR	95% CI		
1	Top	Banana	28 cm	33.63 ± 33.87	1.00	1.00	90.63%	4.24 ± 2.11
2	Top	Banana	26 cm	31.91 ± 31.82	1.05	0.60–1.83	93.75%	4.34 ± 2.33
3	Middle	Banana	28 cm	30.10 ± 18.73	1.01	0.63–1.62	96.88%	4.62 ± 1.94
4	Middle	Banana	26 cm	31.06 ± 24.59	1.04	0.68–1.59	93.75%	4.48 ± 2.12
5	Top	Straight-to-cuff	35°	34.81 ± 27.70	0.87	0.55–1.37	93.75%	4.67 ± 2.30
6	Top	Straight-to-cuff	50°	28.03 ± 16.36	1.11	0.68–1.82	96.88%	4.36 ± 2.32
7	Middle	Straight-to-cuff	35°	34.10 ± 32.12	1.00	0.65–1.53	93.75%	4.29 ± 4.48
8	Middle	Straight-to-cuff	50°	27.53 ± 16.31	1.15	0.74–1.78	96.88%	3.90 ± 2.12
9	Lifting of epiglottis	Straight-to-cuff	35°	23.75 ± 14.24	1.54	1.01–2.34	100%	4.15 ± 2.22
10	Lifting of epiglottis	Straight-to-cuff	50°	20.72 ± 6.90	1.85	1.23–2.78	100%	3.97 ± 2.13

**Fig. 3:**
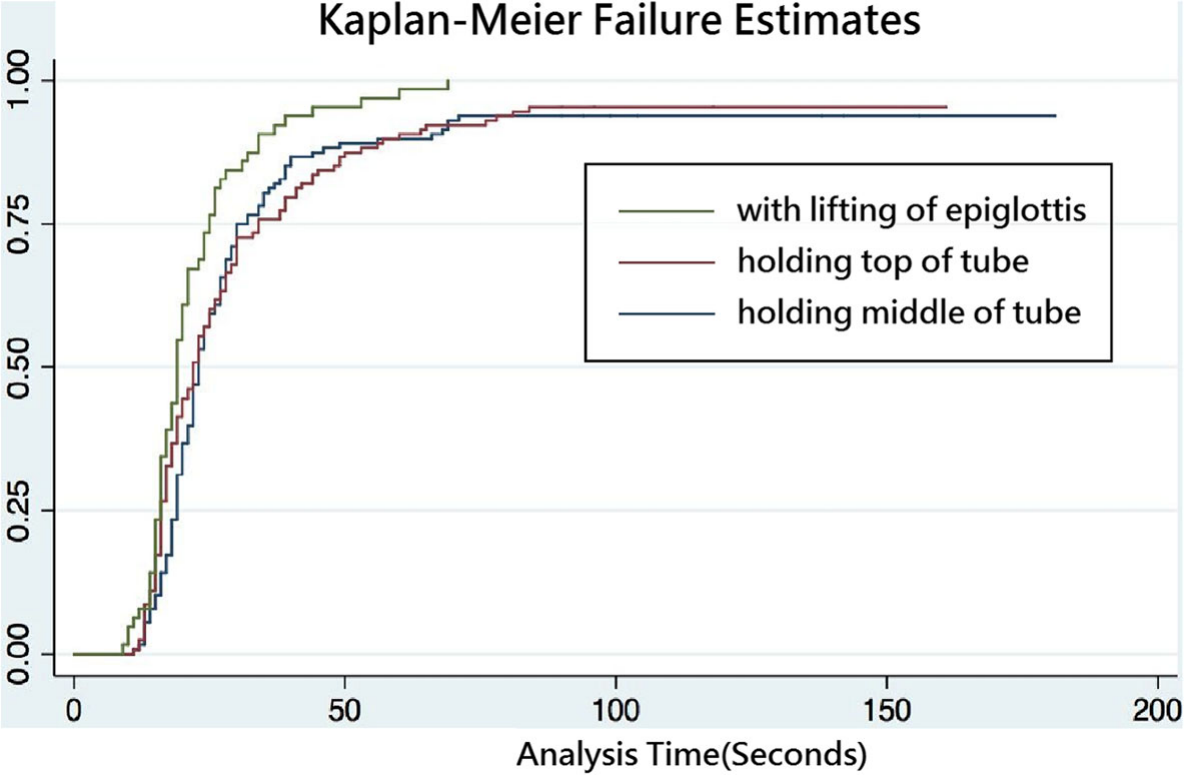
Kaplan–Meier failure estimate for different intubation postures: lifting the epiglottis was more successful than holding the top or middle of the endotracheal tube (*p* < 0.001)

A Kaplan-Meier plot (Fig. 3) illustrates a significantly shorter time to successful intubation with the two subgroups that performed lifting of the epiglottis with stylets (23.75 ± 14.24 s and 20.72 ± 6.90 s among bend angles of 35° and 50°, respectively) and 100% success rate of intubation. In the Cox regression analysis, only the lifting of the epiglottis was significant (*p* < 0.0001, Table 3).

**Table 3:** Analysis of the results of covariates

Variable	HR	95% CI	*p*-Value
Shapes of stylet (banana vs. straight-to-cuff)	0.99	0.82–1.20	0.950
Lifting of epiglottis	1.68	1.34–2.11	< 0.001
Holding level of tube (middle vs. top)	1.04	0.86–1.26	0.675
Duration of service	1.00	0.96–1.04	0.945
Height of the participant	1.09	0.97–1.05	0.631

Based on the results from logistic and linear regression models, VAS was strongly related to intubation time. For each 1 cm increase in the VAS, the odds ratio of success was increased by 0.55, and the intubation time increased by 3.77 s. (Tables 2 and 3, respectively).

## Discussion

Levitan et al. found that for bend angles beyond 35°, straight-to-cuff ET can jeopardize the success rate of tracheal intubation and increase the difficulty of passing the ET [4]. However, among difficult airway, such as C-L III, larger angulations might confer some advantage. In our study, the straight-to-cuff and bend angles at 50° exhibited a trend of shorter intubation times and better success rates compared to bend angles at 35°, despite the method of holding the ET parts (97.92% versus 95.83%, 25.43 ± 14.17 versus 30.89 ± 26.05 s, *p* = 0.0859, 95% CI 0.97–1.61, respectively). Participants also felt that intubation was easier if a larger angulation was used (4.08 ± 2.18 versus 4.37 ± 2.20 cm). On the contrary, banana-shaped ET with larger angulations (longitudinal distance of 26 versus 28 cm) did not show any difference in the success rate, intubation time, or VAS score (equally 93.75%, 31.48 ± 28.21 versus 31.86 ± 27.21 s, 4.41 ± 2.21 versus 4.43 ± 2.02 cm, respectively). The banana shape might cross the visual axis twice if the tube axis is parallel to the visual axis. Thus, this method is believed to be unfavorable for intubation. Our results, however, showed no difference between the straight-to-cuff and banana-shaped tubes despite the effect of the bend angles and holding parts of the tube on success rate, intubation time, and subjective difficulty (96.88% versus 93.75%, 28.16 ± 21.10 versus 31.67 ± 27.60 s, 4.22 ± 2.19 versus 4.42 ± 2.11 cm, respectively).

By holding the top of the ET and flexing the elbow to the chest when the cuff passes the incisors, a larger torque is generated, which might be useful in managing a difficult airway. Compared to holding the middle part of the endotracheal tube, the tilting angle of the ET tip is larger during intubation when holding the top and starting up by the elbow instead of the wrist. However, no significant difference was found between holding the top or holding the middle of the ET on intubation success rates or VAS scores in spite of different bend angles and shapes (32.09 ± 28.04 versus 27.88 ± 20.66 s; 93.75% versus 96.88%; 4.40 ± 2.25 versus 4.23 ± 2.10 cm, respectively). The participants in our study were all inexperienced in holding the ET on the top before our study. Such a condition may lead to a higher failure rate. However, our study showed no evidence that the holding position improved the outcome of the management of the difficult airway.

When only the epiglottis is visible under laryngoscopy (C-L grade III), the ET passing between the vocal cord and trachea cannot be visualized directly and therefore, is considered a blind intubation. The success rate in the first attempt of intubations in C-L grade III was only 44.7% under direct laryngoscopy in one report in an ED setting [5]. When an intubation attempt fails, the practitioner usually will reposition the patient and focus on the left hand go deeper toward the vallecula or lift harder to obtain a better glottic view. However, we often neglect our right hand, which is directly lifting the epiglottis with stylets. This type of manoeuvre can improve the glottic view and allow the practitioner to focus only on the left hand. Nestling the tip of the endotracheal tube under the epiglottis and moving it along the anterior larynx leads to the tracheal inlet. Ueda et al. reported a case wherein lifting of the epiglottis with stylets helped improve the glottic view [3]. In our study, the success rate of intubation with stylet-assisted epiglottis lifting was 100% in contrast to without lifting (94.53%). Lifting of the epiglottis also decreased the mean duration of intubation more than without lifting (22.23 ± 11.20 versus 31.39 ± 25.86 s, respectively). In the survival analysis, lifting of the epiglottis was a strong factor in improving intubation (Fig. 3, *p* < 0.0001). Additionally, lifting the epiglottis with stylets was not considered to be more difficult compared to not lifting the epiglottis (4.06 ± 2.16 cm versus 4.36 ± 2.16 cm, *p* = 0.137, 95% CI -0.80-0.11). Lifting of the epiglottis can accelerate intubation and improve the success rate in difficult intubations without increasing the level of difficulty.

Among the different conditions associated with C-L grade III in our study, holding the top of the endotracheal tube with a straight-to-cuff tube and a bend angle of 35° was considered to be the most difficult intubation performed, followed by holding the middle of the tube with the smaller curvature of the banana-shape (longitudinal distance = 28 cm). In contrast, holding the middle of the ET with the straight-to-cuff tube and a bend angle of 50° was the easiest. The VAS score was directly related to intubation time (odds ratio = 0.55), such that the higher the VAS scores, the longer the intubation duration will be.

### Limitations

First, this was a mannequin study. During the intubations, the participants were asked to avoid better glottic visualisation and continue intubating on a simulated C-L defined difficult airway. Such conditions are likely to not occur in the clinical setting. However, we recorded the entire intubation process on video clips and reviewed them with respect to the precise time intervals and C-L grades just before intubation. We found that the participants tended to lift the epiglottis unintentionally, particularly when they repeatedly failed to pass the ET. However, in simulating the clinical setting, prohibiting contact with the epiglottis would not be natural. Although the study was designed to investigate C-L grade III where only the epiglottis was visible, some of the glottic views, as reviewed by the video clips, were grade IIb (3.13%), in which the lower part of the vocal cord might be seen. The inexperience of the participants in ‘holding the top’ of the ET technique might have reduced the significance of the result in this group. Moreover, the study results were based on direct laryngoscopy on mannequin, further study need to be investigated for the video laryngoscopy and safety on real patients. Finally, this was a convenience sample and may be vulnerable to selection bias. But this study focused on experienced intubation performers that managing difficult airway in their daily practice and the technique of epiglottis lifting still significantly accelerated the intubation process despite their insufficient practice of this new technique.

## Conclusions

When tested on a manikin, lifting the epiglottis with stylets as an adjunctive technique can facilitate and improve the success rate of intubation in difficult airway, such as C-L III, under direct laryngoscopy without increasing the difficulty of the intubation. Our study suggests that based on proper laryngoscope management, using the tip of the stylet-equipped ET to lift the epiglottis and following the anterior part of larynx can significantly facilitate tracheal intubation where only the epiglottis is seen.

## Abbreviations

C-L: Cormack-Lehane
ED: emergency department
ET: endotracheal
HRs: hazard ratios
VAS: visual analogue scale
